# Quetiapine in refractory hyperactive and mixed intensive care delirium: a case series

**DOI:** 10.1186/cc10294

**Published:** 2011-06-28

**Authors:** Ruth YY Wan, Moneesha Kasliwal, Catherine A McKenzie, Nicholas A Barrett

**Affiliations:** 1 Institute of Pharmaceutical Sciences, Kings College London and Department of Pharmacy Guy's and St Thomas' NHS Foundation Trust, Westminster Bridge Road, London SE1 7EH, UK; 2 Department of Critical Care, Guy's and St Thomas' NHS Foundation Trust, Westminster Bridge Road, London SE1 7EH, UK

## Abstract

**Introduction:**

Delirium affects up to 80% of patients admitted to intensive care units (ICUs) and contributes to increased morbidity and mortality. Haloperidol is the gold standard for treatment, although quetiapine has been successfully used in the management of delirium.

**Methods:**

We conducted a retrospective study of patients admitted to the ICU between February 2008 and May 2010 who were prescribed quetiapine by the attending clinician. Data collected included demographics, history of drug and/or alcohol dependence, ICU and hospital length of stay, length of mechanical ventilation and the duration of treatment with sedatives and medications for delirium. The daily dose of quetiapine was recorded. Hyperactive or mixed delirium was identified by a validated chart review and a Richmond Agitation Sedation Scale (RASS) score persistently greater than 1 for 48 hours despite therapy.

**Results:**

Seventeen patients were included. Delirium onset occurred after a median of five days. Patients were being given at least four agents for delirium prior to the introduction of quetiapine, and they had a median RASS score of 3. Quetiapine was initiated at a 25 mg daily dose and titrated to a median daily dose of 50 mg. The median duration of delirium prior to quetiapine therapy was 15 days. Quetiapine commencement was associated with a reduction in the need for other medications (within 0 to 6 days) and resolution of delirium within a median of four days. Adverse events included somnolence and transient hypotension.

**Conclusions:**

This case series provides an initial effort to explore a possible role for quetiapine in the management of refractory hyperactive and mixed ICU delirium.

## Introduction

Delirium is an acute, reversible and fluctuating alteration in consciousness and mentation. It is reported in up to 33% of acutely hospitalised patients [1] and in 20% to 80% of patients admitted to the intensive care unit (ICU) [2-6]. Delirium may manifest as a reduction in the level of consciousness with increased somnolence, decreased cognition and responsiveness or as a state of hypervigilance, agitation and confusion. Delirium may also fluctuate between these two extremes. Delirium has a significant impact upon patients within the ICU, and its presence has been associated with increased mortality, increased length of ICU and hospital stay, increased length of ventilator dependence and increased sedation use [2,7-9]. Delirium is also a significant risk factor for the development of post-ICU cognitive dysfunction, including posttraumatic stress disorder [10]. Multiple factors contribute to the development of delirium, including preexisting cognitive dysfunction, alcohol and/or drug withdrawal, sedative use, altered or inadequate sleep, painful procedures, lack of a focal point, infection, shock states and disordered physiology (for example, hypercapnoea or hypoxia) [10,11].

Managing patients with delirium can be extremely challenging. The optimal management of delirium requires a calm environment, sleep, good hygiene, correction of any underlying contributing factors and minimising sedative use [12]. These can be challenging in the ICU, given the environment and the patient acuity. Agitated patients may cause harm to themselves by inadvertently removing endotracheal tubes, venous access or invasive monitoring. Hence, to preserve patient safety, drug therapy including haloperidol, clonidine, benzodiazepines or propofol is commonly used.

Haloperidol has remained the gold standard of drug therapy for ICU delirium, despite significant limitations due to adverse events, including QTc prolongation (with the potential to trigger ventricular tachycardia, especially Torsades de Pointes), hypotension and extrapyramidal side effects. Haloperidol's status as the gold standard for delirium therapy is predominantly a result of historical precedent [13]. However, some investigators have reported that haloperidol may prolong delirium in the ICU population [14-16]. To ensure maximum effect and minimise adverse events, it has been advised that haloperidol should be administered in small, frequent doses (0.5 to 1 mg every two to four hours) [17,18]. This can be difficult to achieve in clinical practice, and thus it is possible that poor administration contributes to delirium and agitation or results in an increase in adverse events as a result of this treatment [19].

Quetiapine, an atypical antipsychotic drug, has demonstrated efficacy in psychiatry and has been used to treat conditions such as acute schizophrenia, mania, depression and bipolar disorder [20]. Recent reports have also demonstrated that quetiapine can be of benefit in the management of delirium in older medical patients [21]. Quetiapine has 'loose' or low binding at D2 receptors, which has been reported to be favourable in delirium management [22,23]. In addition, quetiapine has several pharmacokinetic properties that make it potentially advantageous in the ICU. It is administered every 12 hours, has a relative short half-life of 7 hours (12 hours for its active metabolite norquetiapine) [19] and is readily titratable, and it causes a lower incidence of QTc prolongation and fewer extrapyramidal symptoms than haloperidol [18,20,24].

In a recent randomised, controlled trial, quetiapine was shown to be efficacious when added to haloperidol in the management of delirium in a tightly defined ICU population [25]. The successful use of quetiapine in refractory hyperactive delirium in the ICU has also been previously reported in a case series of five patients [26]. It has been the usual practice in our institution to administer quetiapine to patients with hyperactive or mixed delirium refractory to other management strategies.

## Materials and methods

This review of a retrospective case series was approved by the Guys and St Thomas' NHS Foundation Trust Clinical Audit Group (registration number 2023). Patient consent was waived, and the review was unfunded. The case series was carried out in a 30-bed level 3 ICU that has more than 1,200 admissions annually. We included the case notes of patients admitted from February 2008 to May 2010 who were prescribed quetiapine by the attending clinician for hyperactive or mixed delirium. Patients were excluded if they were taking quetiapine for a preexisting psychiatric illness. The data collected included demographic data, admitting diagnosis, drug and/or alcohol dependence, ICU and hospital length of stay, length of mechanical ventilation, the duration of sedatives and/or opioids to control agitation, and support ventilation and other therapies used to specifically manage delirium or agitation (haloperidol or clonidine). The dose of quetiapine at the initiation of therapy and subsequent dose changes were recorded. Adverse effects commonly observed during quetiapine therapy were recorded, and these included sedation, hypotension, extrapyramidal side effects and QTc prolongation [20]. The median daily Richmond Agitation Sedation Scale (RASS) score was calculated, and a validated retrospective chart review was performed to identify delirium [27]. Refractory hyperactive or mixed delirium was retrospectively identified if the patient's RASS score was greater than 1 for more than 48 hours, the daily validated chart review indicated the presence of an acute confusional state (for example, delirium, mental status change, inattention, disorientation, hallucinations, agitation or inappropriate behaviour) and two or more agents were used prior to the introduction of quetiapine known to treat delirium. The validated chart review was undertaken by a junior doctor, and the results were confirmed by a senior clinical pharmacist.

## Results

A total of 19 patients were prescribed quetiapine, 2 of whom were excluded because quetiapine had been prescribed for chronic psychiatric indications; hence 17 patients were included in the case series (Table 1). Fifteen patients were male with a mean patient age of 59 years. Eight patients had a history of drug or alcohol abuse. The validated chart review confirmed in all cases; alcohol or drug withdrawal was not believed to be the cause of delirium as viewed by the attending clinician.

**Table 1:** Patient characteristics^a^

Characteristics	Data
Number of patients	17
Mean age, years (range)	59 (30 to 85)
Males, *n *(%)	15 (88%)
Number of ventilated patients, *n *(%)	16 (94.1%)
Tracheotomy	12 (75%)
Intubated	4 (25%)
Median total length of stay in ICU, days (range)	41 (16 to 235)
Location before ICU, *n *(%)	
Home	9 (52.9%)
Another hospital	4 (23.5%)
Ward	4 (23.5%)
Case mix, *n *(%)	
Medical	10 (59%)
Surgical	7 (41%)
Admission diagnosis, *n *(%)	
Sepsis/acute respiratory distress syndrome	8 (47%)
Surgery	4 (23.5%)
Myocardial infarction	4 (23.5%)
Other	1 (6%)
Alcohol excess, *n *(%)	6 (35.2%)^b^
Substance abuse, *n *(%)	2 (11.7%)^b^
Median days to delirium onset after admission to ICU, *n *(range)	5 (0 to 20)

^a^ICU, intensive care unit. ^b^One patient had a history of substance abuse and alcohol excess.

Prior to the introduction of quetiapine, the patients had a median RASS score of 3 and were being treated with two or more drugs for delirium, and there was consistent documentation of an acute confusional state identified in the validated chart review. The median time until delirium onset was day 5 from ICU admission (Table 1), and the median duration of refractory hyperactive or mixed delirium was 15 days prior to the introduction of quetiapine (Table 2). Prior to the commencement of quetiapine therapy, all patients had been taking four or five agents to control their delirium and agitation (Table 2).

**Table 2:** Clinical outcomes and relevant medication therapy

Outcomes	Median pre-quetiapine days, *n *(range)	Median post-quetiapine days, *n *(range)
Duration of refractory delirium	15 (3 to 48)	4 (2 to 29)
Duration of ventilatory support	20.5 (1 to 58)	11 (3 to 17)
Duration of ventilatory support since RASS score 0 achieved	-	2 (1 to 11)
Haloperidol *n *= 14	6 (1 to 37)	0.5 (0 to 7)
Lorazepam, *n *= 10	9.5 (1 to 19)	1 (0 to 7)
Propofol, *n *= 17	17 (5 to 27)	6 (0 to 18)
Opioids (fentanyl/alfentanil), *n *= 17	18 (7 to 34)	0 (0 to 16)
Clonidine, *n *= 14	9.5 (2 to 33)	2 (0 to 16)

Quetiapine was initiated by either the oral or enteral route at a daily dose of 25 mg (normally administered in 12-hourly divided doses) for all patients, and the dose was titrated by the attending clinician. The median total daily dose prescribed was 50 mg (range, 12.5 to 400 mg). Following the introduction of quetiapine, the patients had a reduced need for other medications and experienced resolution of refractory hyperactive or mixed delirium within a median of four days (Table 2).

Adverse effects were recorded in four patients. One patient developed QTc prolongation, which was defined as an increase of more than 60 milliseconds from baseline. Excessive somnolence was noted in one patient, and transient hypotension was noted in the two remaining patients but was not significant enough to warrant discontinuation of quetiapine. No extrapyramidal symptoms were observed.

Quetiapine was ceased in 10 patients before they left the ICU. In four patients, it was ceased on the ward following discharge from the ICU. One patient was discharged to home, and two were discharged to their local hospitals prior to cessation of quetiapine. When or if they stopped taking quetiapine is not known.

## Discussion

In this retrospective case series, we found that there is a temporal association between the commencement of quetiapine therapy and the resolution of refractory hyperactive or mixed delirium. Patients had been in a hyperactive or mixed delirium for a median of 15 days prior to quetiapine initiation, but this state resolved within a median of 4 days after the start of quetiapine treatment. Once quetiapine therapy was commenced, patients were weaned rapidly from most other medications over a median of zero to two days, with propofol treatment continued for a median of six days. The patients who were given quetiapine were considered to have refractory mixed or hyperactive delirium and required the attending clinicians to use challenging management strategies. Quetiapine was well tolerated, with transient hypotension, somnolence and asymptomatic QTc prolongation recorded for only four patients. Quetiapine was ceased in 10 patients before they left the ICU and in another 4 patients before they left the hospital. Given that this is an uncontrolled case series, these data cannot provide proof of efficacy. However, it does encourage the development of randomised, controlled trials of quetiapine in this patient population.

The patients who received quetiapine in this case series are recognised as the group at the highest risk of developing hyperactive or mixed delirium which is refractory to treatment [7]. In particular, this series contained mixed medical and surgical patients who predominantly presented with sepsis or severe respiratory failure and included a high proportion of patients with drug and/or alcohol dependence. A recent randomised, controlled trial in ICUs that demonstrated the benefits of quetiapine therapy in patients with delirium demonstrated resolution of delirium within a median of one day [25]; however, that study was performed in a more homogeneous patient group with a shorter duration of delirium and fewer risks for refractory delirium than the current case series. It is therefore encouraging that the delirium in the current series resolved soon after the commencement of quetiapine. This could potentially be associated with the administration of quetiapine itself, the concomitant reduction in opioids and other sedatives or by chance alone [7].

The quetiapine doses used in our case series were initially lower than those reported in the recent study by Devlin and colleagues [25], which commenced at a total of 100 mg daily administered in 12-hourly divided doses. The median maximum total daily dose of 50 mg reported in our case series is also lower than those in other studies, in which median maximum total daily doses of 400 mg were used [28]. The two patients who received a total daily dose greater than 200 mg in our series had a history of alcohol dependence, which is known to require an increased dose of psychotropic medications [6]. Although the results of the current series are not conclusive, it is encouraging that lower doses could be prescribed, which may provide guidance for future studies.

Few adverse events were noted overall in the case notes of these patients. Problems were predominantly related to somnolence and postural hypotension, which are similar to those described in the literature [20,24]. Despite the presence of hypotension, quetiapine treatment was continued as the attending clinician considered that other causes for hypotension were present and the hypotension resolved spontaneously. One patient developed a prolonged QTc interval, which resulted in discontinuation of the drug. This patient was receiving concomitant fluconazole, an inhibitor of the cytochrome P450 isoenzyme CYP3A4 that also metabolises quetiapine. Concurrent use of fluconazole increases quetiapine levels, thus the QTc prolongation could be considered the result of a drug interaction rather than an adverse effect [29]. No episodes of extrapyramidal side effects were noted; however, an extrapyramidal side effect rating scale is not routinely used in our institution. The retrospective assessment of adverse events can be very difficult, and it is likely that adverse events were underrecognised, especially given that the rate of adverse events was lower than the rates reported in the literature [28,30].

In this case series, the median daily RASS score, use of more than two medications and a validated case note review were used to evaluate hyperactive or mixed delirium. The use of a validated case note review is an accepted method of establishing the clinician's opinion that a state of delirium exists, although considerable expertise is required to undertake it [27]. In this case series, the validated chart review was undertaken by a junior doctor and was then confirmed by a senior clinical pharmacist in all cases. The RASS is a subjective 11-point scale that has discrete criteria for levels of sedation and agitation. It has been reported to have high reliability and validity for sedation and agitation in medical, surgical, ventilated, nonventilated, sedated and nonsedated ICU patients and is commonly used in the UK [31,32]. The RASS score is routinely measured hourly, providing a longitudinal measure of agitation and sedation [33]. The RASS is not a delirium scoring system, and agitation may be due to a number of reasons (including pain or physiological dysfunction). However, agitation is a common feature of hyperactive or mixed delirium, and the combination of the RASS score with the validated case note review should identify patients in whom delirium is believed to exist by the attending clinician. Specific delirium scoring systems such as the confusion assessment method (CAM)-ICU have a higher reported sensitivity and specificity and higher interrater reliability (κ = 0.96) for the assessment of delirium [34]. However, our ICU, like many in the UK, does not use CAM-ICU or any other dedicated delirium screening checklist (for example, the intensive care delirium screening checklist (ICDSC)) in routine clinical practice [32].

This study comprised a small, uncontrolled, retrospective, single-centre case series and has significant potential limitations. Selection bias is clearly a problem, given that patients received quetiapine on the basis of the treating physician's opinion. The cohort of patients was small, and no control group was used. Although the data suggest a temporal relationship between the initiation of quetiapine therapy and the resolution of delirium, no causal relationship can be established. It is entirely possible that the patients' delirium was resolving at the same time that quetiapine was commenced. Follow-up was incomplete, and no formal neuropsychological assessment was made following discharge from the ICU; hence no comment can be made regarding the longer-term sequelae of delirium and posttraumatic stress disorder. Given the retrospective, uncontrolled nature of the review, other unrecognised sources of bias might exist. All of these methodological weaknesses could be addressed by a suitably powered, prospective, randomised, placebo-controlled trial. Any prospective evaluation of delirium should include validated tools (ICDSC or CAM-ICU) to support the diagnosis of delirium and the possible effects of quetiapine in the treatment of delirium.

## Conclusions

In this article, we describe a retrospective case series in which we studied the clinical course of patients with difficult-to-manage hyperactive or mixed delirium who received quetiapine at the request of the attending clinician. The commencement of quetiapine treatment might be temporally associated with the resolution of hyperactive and mixed delirium and a reduction in sedative dose. Nonetheless, the absence of controls does not allow us to draw any definitive conclusions regarding the effects of quetiapine in patients with hyperactive and mixed delirium. The low doses used and the low adverse event rate in our case series will be helpful by providing guidance for future prospective trials.

## Key messages

• Quetiapine might be an effective therapy in difficult-to-treat patients with hyperactive and mixed delirium.

• Quetiapine may be efficacious at doses lower than those reported in the literature.

• Quetiapine's effectiveness in this patient group should be tested further in a controlled study.

## Abbreviations

CAM-ICU: Confusion Assessment Method-Intensive Care Unit; ICDSC: Intensive Care Delirium Screening Checklist; ICU: intensive care unit; RASS: Richmond Agitation Sedation Scale.

## Competing interests

The authors declare that they have no competing interests.

## Authors' contributions

NB and CM devised and supervised the case review. MK and RW conducted the review. All authors contributed to the manuscript. All authors read and approved the final version of the manuscript.

## Authors' information

NB is consultant intensivist, and CM is a consultant pharmacist practising in intensive care. They have an interest in sedation and delirium management in critically ill patients. RW is a senior clinical pharmacist in critical care, and MK is a trainee intensivist.
